# A histamine-gated channel is an efficient negative selection marker for *C. elegans* transgenesis

**DOI:** 10.17912/micropub.biology.000349

**Published:** 2021-01-08

**Authors:** Sonia El Mouridi, Sarah AlHarbi, Christian Frøkjær-Jensen

**Affiliations:** 1 King Abdullah University of Science and Technology (KAUST), Biological and Environmental Science and Engineering Division (BESE), KAUST Environmental Epigenetics Program (KEEP), Thuwal, 23955-6900, Saudi Arabia

**Figure 1. The histamine-gated chloride channel HisCl1 effectively paralyzes transgenic array animals:**
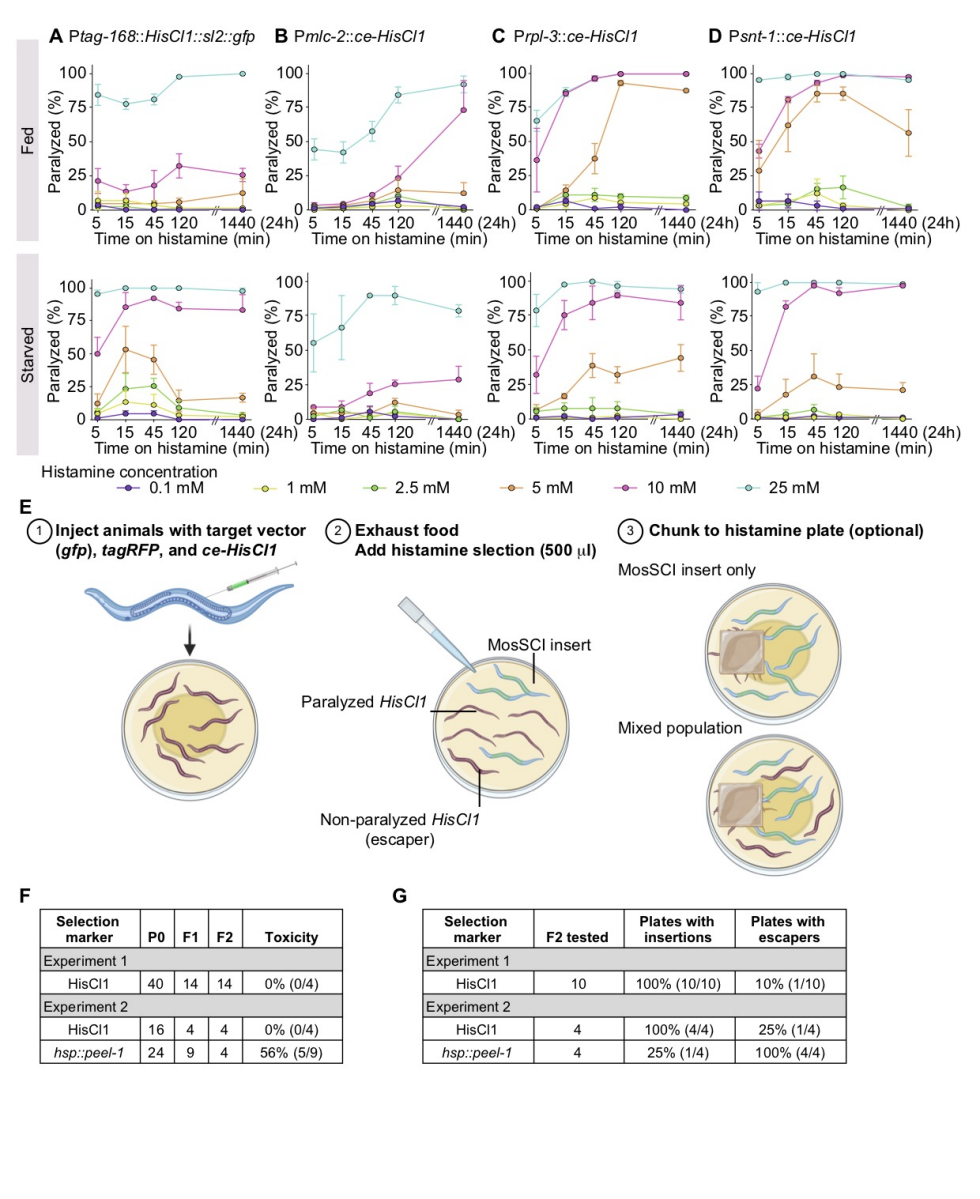
**A-D.** Dose-response relationship and time-course for the paralysis of transgenic animals with *HisCl1* expressed from the indicated promoters in response to increasing histamine concentrations and longer exposure. P*tag-168* primarily drives expression in the nervous system. P*mlc-2* is pan-muscular (El Mouridi *et al.*, 2020), P*rpl-3* is a strong ribosomal promoter (likely ubiquitously expressed), and P*snt-1* is widely expressed in the nervous system (Nonet *et al.*, 1993). Top panels: fed animals on OP50 bacteria. Bottom panels: starved L1 animals. Quantification: Four plates with 15 transgenic animals were scored by eye for paralysis for each promoter and condition (concentration and time-point) in two technical replicates. **E.** Schematic showing the use of histamine selection to identify Mos1-mediated Single-Copy transgene Insertions (MosSCI) (Frøkjær-Jensen *et al.*, 2008) using a red marker to identify arrays (P*mlc-1*::tagRFP-T), and a plasmid expressing histamine (P*snt-1*::*ce-HisCl1*) as a negative selection marker. Insertions were identified from starved plates with histamine or by “chunking” onto plates with histamine. **F.** Quantification of relative toxicity of histamine and *hsp*::*peel-1* selection. We used the frequency of stable F2 rescued lines relative to the number of plates with F1 rescue as a proxy for toxicity. **G**. Quantification of the MosSCI insertion frequency and the frequency of plates with “escapers” (moving animals with fluorescent array markers).

## Description

Gene edits are often recovered at high frequency at the population level but only infrequently in individual animals. For example, a single injected *C. elegans* has on the order of 10,000 F2 progeny, but only a small fraction of these animals will carry the desired genetic modification. Thus, a key challenge for efficient transgenesis is developing methods to easily identify these rare individuals in a sea of wild-type animals. Therefore, positive and negative (or counter-) selection markers play an essential role in gene editing. Positive selection markers confer an advantage to the organism, *e.g.*, the phenotypic rescue of mutant phenotypes or antibiotic resistance. In *C. elegans*, commonly used phenotypic rescue markers include *lin-15* (Clark *et al.*, 1994), *pha-1(ts)* (Granato *et al.*, 1994), and *unc-119* (Maduro and Pilgrim, 1995) or the antibiotic resistance markers *hygroR* (Radman *et al.*, 2013), *NeoR* (Giordano-Santini *et al.*, 2010), *PuroR* (Semple *et al.*, 2010), and *BsrR* (Kim *et al.*, 2014). In contrast, negative selection markers can be used to avoid false-positive animals that carry positive selection markers but no genetic modification. Such selection is particularly important for transgenic methods based on plasmid injection because extra-chromosomal arrays are often formed and propagated as an intermediate step. For example, targeted insertion of transgenes by MosSCI (Frøkjær-Jensen *et al.*, 2008) and CRISPR (Dickinson *et al.*, 2013) or random transgene insertion by miniMos transposition (Frøkjær-Jensen *et al.*, 2014) all initially establish array lines and subsequently identify edits in animals that have lost the array. Only a few negative selection markers are commonly used. Fluorescent co-injection markers can be used to identify array animals but do not exert selective pressure and require visual screening on a fluorescence microscope. An alternative negative selection strategy relies on inducible expression of a toxin, *peel-1*, to kill animals with arrays (Frøkjær-Jensen *et al.*, 2012; McDiarmid *et al.*, 2020; Seidel *et al.*, 2011). *peel-1* based selection is easily scaled to large populations (*i.e.*, many individual plates) by simple heat-shock. However, the selection has several drawbacks. First, *peel-1* is toxic in the absence of heat-shock, which reduces the efficiency of establishing transgenic array animals (Frøkjær-Jensen *et al.*, 2012). Second, the selection is relatively slow because it takes several hours for the toxin to kill animals, and screening is best done the day after heat-shock. Third, and more frustrating, *peel-1* selection is often not fully penetrant, and heat-shocked plates frequently contain false positive “escapers”, *i.e.*, array animals that are not killed by the toxin. Therefore, it would be beneficial to have additional negative selection methods to complement or substitute for *peel-1* based selection.

Here, we describe an efficient negative selection method based on the addition of histamine to plates. Histamine is inexpensive and has modest toxicity allowing routine use in the laboratory. In many animals, histamine is used as a signaling molecule in the immune system. In *D. melanogaster,* endogenous histamine functions as a neurotransmitter by binding to inhibitory histamine-gated chloride channels (*HisCl1*). Pokala *et al.* (2014) used the lack of endogenous histamine signaling in *C. elegans* to develop a method for inducible silencing of neurons by cell-specific expression of *HisCl1*. In this system, animals with pan-neuronal expression of *HisCl1* are fully paralyzed by exogenous addition of histamine. Including *HisCl1* in arrays could, therefore, possibly be used as a negative selection strategy. There is some precedence for using histamine for selection: positive hygromycin selection has been combined with negative histamine selection (“HyHis-Cl”). This approach was used to delete a gene by CRISPR and subsequently excise the selection cassette with Cre recombinase, although the authors provided few experimental details (*e.g.*, drug concentration or selection protocol) (Abiusi *et al.*, 2017).

Motivated by these studies, we generated reagents and tested histamine paralysis under conditions compatible with extra-chromosomal arrays and standard protocols for transgene insertion. First, we optimized *HisCl1* expression for *C. elegans* (*ce-HisCl1*) and generated expression constructs that lack a fluorescent co-expression marker. pNP403 (P*tag-168::HisCl1::sl2::gfp*), used by Pokala *et al.* (2014), co-expresses *gfp* in neurons, the intestine, and some muscles, which interferes with easy screening for transgenes with *gfp* tags. We expressed the optimized *ce-HisCl1* under a pan-muscular promoter (P*mlc-2*) (El Mouridi *et al.*, 2020), a pan-neuronal promoter (P*snt-1*) (Nonet *et al.*, 1993), and a strong ribosomal promoter (P*rpl-3*). Second, for histamine selection to be practical, we would ideally add histamine directly to starved plates (the easiest stage to screen for transgene insertions), and the paralysis should be non-reversible. We performed dose-response experiments by adding 500 ml histamine directly to plates with transgenic animals on food (OP50 bacteria) (**Figure 1A-D, top**) or to plates with starved transgenic animals (**Figure 1A-D, bottom**). We generated three independent transgenic array lines for each of the four promoters and tested six different histamine concentrations (0.1 mM, 1 mM, 2.5 mM, 5 mM, 10 mM, and 25 mM) with short (5, 15, and 45 min) or long (2 and 24 hours) exposure times. We observed infrequent paralysis at low histamine concentrations (0.1 to 5 mM) but penetrant paralysis at higher concentrations (10 to 25 mM) using all four promoters (**Figure 1 A**–**D**). Codon-optimization modestly improved the constructs but we observed limited effects from expression in muscles, possibly because ectopic chloride channel expression is not enough to depolarize the relatively large muscle cells. Intermediate histamine concentrations (*e.g.*, 5 mM) were sensitive to differences between fed and starved plates. In contrast, higher histamine concentrations (10 to 25 mM) resulted in rapid, non-reversible paralysis both on and off food (**Figure 1** **A-D**).

We tested a simple selection scheme with P*snt-1::ce-HisCl1* (pSEM238) as a negative selection marker to generate single-copy transgene insertions (**Figure 1** **E**). First, we injected 40 animals (EG6699: *ttTi5605* II, *unc-119*(*ed3*) III) with a mix of the target vector (P*smu-1*::*gfp*), a fluorescent co-injection marker (P*mlc-1*::TagRFP-T) (El Mouridi *et al.*, 2020), and P*snt-1::ce-HisCl1*. We placed a single injected animal on individual NGM plates and recovered 14 plates with stable extra-chromosomal lines based on Unc-119 rescue (**Figure 1 F-G**). Notably, all plates with F1 rescued animals gave stable F2 lines, suggesting that inclusion of the histamine selection plasmid did not reduce transgenesis efficiency. From these F2 lines, we selected ten plates for testing the negative histamine selection. We added 500 ml of histamine (500 mM) to ten starved plates and screened for putative MosSCI insertions using two approaches (in both cases blinded to the fluorescence of transgenic animals). We either picked three non-paralyzed animals directly from the starved plate with histamine after 15 minutes or chunked a small part of the plate to a new plate with food and histamine (**Figure** **1E**). Both approaches gave identical results: we were able to isolate MosSCI insertions from all ten plates, and only one plate had a mix of moving animals with insertions and arrays. In a second experiment, we directly compared the efficiency of histamine relative to *hsp*::*peel-1* selection (**Figure 1F-G**). As previously observed (Frøkjær-Jensen *et al.*, 2012), inclusion of *peel-1* appears to be moderately toxic with a reduced frequency of stable array formation (56% of plates with F1 rescue did not give stable F2 lines) (**Figure 1F**). Furthermore, we observed a higher MosSCI frequency (100% versus 25%) and a lower frequency of false positive escapers (25% versus 100%) when using histamine compared to *peel-1* selection (**Figure 1G**).

Here, we have demonstrated that histamine is useful for identifying single-copy transgene insertions, but we imagine that histamine can also be used in other experiments, such as genetic suppressor screens (Joseph *et al.*, 2018) or for CRISPR-based selections (McDiarmid *et al.*, 2020). In sum, these results demonstrate that histamine is a cheap and effective chemical that can be used to select against array animals.

## Methods

**Molecular biology**

All plasmids were generated using three-fragment multisite Gateway reactions (ThermoFisher) and validated by restriction digest. *ce-HisCl1* was optimized for expression in *C. elegans* by including two early introns (from *ama-1*) which has been shown to increase gene expression (Aljohani *et al.*, 2020). We also minimized the ribosomal binding site energy but did not codon-optimize the *ce-HisCl1* transgene. *ce-HisCl1* was generated by gene synthesis (Twist Biosciences, CA, USA) in a [1-2] Gateway Entry vector.

**NGM plates with histamine (NGM-HA)**

We maintained animals on Nematode Growth Media (NGM) plates seeded with *Escherichia coli* OP50 using standard techniques (Brenner, 1974). We made selective plates by adding histamine directly to NGM plates by pipetting 500 ml of histamine solutions in Milli-Q water (Gold Biotechnology, cat. no. H-110-100) directly onto the bacterial lawn. We assumed that NGM plates contained approximately 10 mL solution and used 500 mM, 200 mM, 100 mM, 50 mM, 20 mM, and 0.2 mM histamine stock solutions. We recommend adding 500 ml of a 500 mM histamine solution to a standard 6 cm NGM plate for routine selection against arrays. Histamine selection costs less than $0.25 per plate.

**Dose-response curve for histamine paralysis**

We generated injection mixes consisting of 10 ng/µl selection plasmid (*Ptag-168::HisCl1::SL2::GFP*, *Pmlc-2::ce-HisCl1*, *Prpl-3::ce-HisCl1*, or *Psnt-1::ce-HisCl1*), 10 ng/µl pSEM233 (*Pmlc-1::TagRFP-T*), 10 ng/µl pCFJ782 (Hygromycin selection), and 70 ng/ml GeneRuler 1kb Plus DNA Ladder (ThermoFisher SM1331) for a final concentration of 100 ng/µl. We injected wild-type (N2) animals and generated three independent lines for each histamine plasmid by selecting for hygromycin resistance. We tested three independent lines of each promoter for histamine-induced paralysis under two conditions: fed and starved. Fed worms were transferred from a plate with OP50 to histamine plates, whereas starved worms were taken from a freshly starved plate with L1 animals. We transferred 15 transgenic animals to NGM-HA plates with 25 mM, 10 mM, 5 mM, 2.5 mM, 1 mM, and 0.1 mM final histamine concentrations and started a timer. We counted paralyzed worms at five different time-points during 24 hours (5 min, 15 min, 45 min, 120 min (2 hours), 1440 min (24 hours)). We performed two technical replicates of the experiments on different days and used wild-type worms as a negative control (we observed no paralysis at any histamine concentration or duration of exposure). The time-course graphs were produced using GraphPad Prism 8 software for macOS.

**MosSCI insertion**

Experiment 1: We generated *Psmu-1::gfp* MosSCI insertions to test the efficiency of *Psnt-1::ce-HisCl1* as a negative selection marker. The injection mix contained 25 ng/µl MosSCI targeting vector (pCFJ1805, P*smu-1*::*gfp*), 25 ng/µl fluorescent co-injection marker (pSEM233, *Pmlc-1::TagRFP-T*), 20 ng/µl of an optimized Mos1 transposase (pCFJ1532, P*smu-1*:*Mos1* transposase), 10 ng/µl negative selection marker (pSEM238, *Psnt-1::ce-*HisCl1), and 20 ng/µl stuffer DNA (GeneRuler 1kb Plus DNA Ladder, ThermoFisher SM1331) for a final DNA concentration of 100 ng/µl.

Experiment 2: We generated *Psmu-1::gfp* MosSCI insertions to compare the efficiency of *Psnt-1::ce-HisCl1* relative to hsp::peel-1 negative selection. The injection mix contained 25 ng/µl MosSCI targeting vector (pCFJ1805, P*smu-1*::*gfp*), 10 ng/µl fluorescent co-injection marker (pSEM233, *Pmlc-1::TagRFP-T*), 20 ng/µl of an optimized Mos1 transposase (pCFJ1532, P*smu-1*:*Mos1* transposase), 20 ng/µl negative selection marker (pMA122, *hsp::peel-1* or pSEM238, *Psnt-1::ce-*HisCl1), and 25 ng/µl stuffer DNA (GeneRuler 1kb Plus DNA Ladder, ThermoFisher SM1331) for a final DNA concentration of 100 ng/µl.

Software

Graphs were produced using GraphPad Prism 8 software for macOS and the schematic was created with Biorender.com.

## Reagents

pNP403 P*tag-168::HisCl1::sl2::unc-54* 3′ UTR (Pokala *et al.*)

pSEM236 P*mlc-2::ce-HisCl1::rpl-3* 3′ UTR (Addgene #159797)

pSEM237 P*rpl-3::ce-HisCl1::rpl-3* 3′ UTR (Addgene #159796)

pSEM238 P*snt-1::ce-HisCl1::rpl-3* 3′ UTR(Addgene #161515)

pSEM233 P*mlc-1*::*tagRFP-T*::*cbr-tbb-2* 3′ UTR (Addgene #159899)

pCFJ1532 P*smu-1*::*mosase(PATC)*::*smu-1* 3′ UTR (Addgene # 159807)

All pSEM vectors are available at Addgene, but we recommend using pSEM238.

Annotated plasmid sequences are available at www.wormbuilder.org and https://addgene.org/Christian_Froekjaer-Jensen/
