# Corrigendum: A histamine-gated channel is an efficient negative selection marker for *C. elegans* transgenesis

**DOI:** 10.17912/micropub.biology.000361

**Published:** 2021-01-25

## Description

For El Mouridi, S; AlHarbi, S; Frøkjær-Jensen, C (2021). A histamine-gated channel is an efficient negative selection marker for *C. elegans* transgenesis. microPublication Biology. 10.17912/micropub.biology.000349.

The authors correct the following:

The units of volume of histamine added to the plates have been corrected from **ml** to **µl** in the following sentences:

In the Description section:

The sentence

“We performed dose-response experiments by adding 500 **ml** histamine directly to plates with transgenic animals on food (OP50 bacteria) (Figure 1A-D, top) or to plates with starved transgenic animals (Figure 1A-D, bottom).”

Is corrected to:

“We performed dose-response experiments by adding 500 **µl** histamine directly to plates with transgenic animals on food (OP50 bacteria) (Figure 1A-D, top) or to plates with starved transgenic animals (Figure 1A-D, bottom).”

The sentence

“We added 500 **ml** of histamine (500 mM) to ten starved plates and screened for putative MosSCI insertions using two approaches (in both cases blinded to the fluorescence of transgenic animals).”

Is corrected to:

“We added 500 **µl** of histamine (500 mM) to ten starved plates and screened for putative MosSCI insertions using two approaches (in both cases blinded to the fluorescence of transgenic animals).”

In the Methods section:

The sentence

“We made selective plates by adding histamine directly to NGM plates by pipetting 500 **ml** of histamine solutions in Milli-Q water (Gold Biotechnology, cat. no. H-110-100) directly onto the bacterial lawn.”

Is corrected to:

“We made selective plates by adding histamine directly to NGM plates by pipetting 500 **µl** of histamine solutions in Milli-Q water (Gold Biotechnology, cat. no. H-110-100) directly onto the bacterial lawn.”

The sentence

“We recommend adding 500 **ml** of a 500 mM histamine solution to a standard 6 cm NGM plate for routine selection against arrays.”

Is corrected to:

“We recommend adding 500 **µl** of a 500 mM histamine solution to a standard 6 cm NGM plate for routine selection against arrays.”

